# Intellectual maturity and longevity: late-blooming composers and writers live longer than child prodigies

**DOI:** 10.18632/aging.101245

**Published:** 2017-05-30

**Authors:** Maurits P.J. Hafkamp, Joris P.J. Slaets, David van Bodegom

**Affiliations:** 1 Leyden Academy on Vitality and Ageing, Leiden, The Netherlands; 2 Department of Internal Medicine, University Medical Center Groningen, Groningen, The Netherlands; 3 Department of Gerontology and Geriatrics, Leiden University Medical Center, Leiden, The Netherlands

**Keywords:** life history theory, intellectual, maturity, longevity, lifespan, development

## Abstract

Life history theory links human physical and sexual development to longevity. However, there have been no studies on the association of intellectual development with longevity. This observational study investigates the relationship between the onset of intellectual maturity and lifespan through the life histories of composers and creative writers, whose intellectual development can be gauged through their compositions and writings. In these groups we model the relationship between the age at first creative work, and age at death using multilevel regression, adjusting for sex, date of birth, and nationality. Historical biographical records on 1110 musical composers and 1182 creative writers, born in the period 1400 AD through 1915 AD, were obtained from the Oxford Companion to Music and the Oxford Companion to English Literature. Composers and creative writers lived, respectively 0.16 (*p* = 0.02) and 0.18 (*p* < 0.01) years longer for each later year of age at first work. When completion of the first creative work is interpreted as a proxy for the onset of intellectual maturity in composers and creative writers, our findings indicate that a later onset of intellectual maturity is associated with higher longevity.

## INTRODUCTION

Life history theory examines relationships between the timings of life events that have relevance for evolutionary fitness [1]. The timing of a life event is itself a reflection of an underlying biological process, such as the process of development in the case of onset of maturity or the processes of repair and maintenance in the case of timing of death. Generally, across animal species, the longer it takes an organism to reach maturity, the longer it lives [2]. How the timing of onset of maturity relates to longevity in humans, is still a matter of debate. In this regard, it is of special interest whether the onset of intellectual maturity is associated with longevity, as the brain and associated parts of the central nervous system comprise an organ system that is widely thought to have played a vital role in humans’ evolutionary success [3].

With respect to the relation between onset of maturity and longevity, it has been observed that women with a later onset of sexual maturity and lower fertility have longer lifespans [4–7]. Similarly, the timing of humans’ physical peak is also correlated with the timing of death: those who peak later, have increased average longevity [8]. However, different organ systems generally develop at different rates and associations that hold true for one system cannot readily be extrapolated to another. Studies into the course of intellectual abilities throughout the lifespan have indeed shown that the course of intellectual development deviates from that of reproductive and physical development [9].

Whereas components of fluid intelligence, e.g. short-term memory, peak around the time when physical maturity is reached, components of crystallized intelligence, e.g. vocabulary, have been shown to peak at ages above thirty or even increase steadily into old age [10–13]. Evolutionarily relevant intellectual capabilities that emerge as a resultant of both fluid and crystallized intelligence might thus have a developmental course that reaches a peak at some intermediate age. However, the aforementioned studies were concerned with characterizing average patterns in cognitive ability across age and made no attempt to correlate individual variation in developmental trajectories with variation in lifespan. Such analyses require longitudinal data that comprise some read-out of an individual's intellectual abilities over time as well as information on lifespan duration. To our best knowledge, no such study has yet been reported.

The present study seeks to investigate the relationship between the onset of intellectual maturity and lifespan through the life histories of composers and creative writers. These groups are appropriate for such an investigation, because they leave publicly accessible records of creative output that reflect individual intellectual capabilities. The first performance of one of a composer's compositions and a writer's first published work could be considered a mark of the onset of an intellectual maturity of sorts, as any work must exhibit a minimum level of intellectual quality to qualify for performance or print and subsequent inclusion in the annals of music and literature. In a historical sample of composers and creative writers, we test the hypothesis that a later onset of intellectual maturity comes with a later age at death.

## RESULTS

As shown in Table 1, 1110 composers and 1182 creative writers respectively made up the two samples on which the analyses described below were carried out. These individuals met the criteria for inclusion in the study: they had been born between the years 1400 and 1915, had lived at least up to the age of forty and had finished a first work before the age of forty. Women made up a small proportion of the sample of creative writers (15.4%), while an even smaller proportion of women was present in the sample of composers (1.2%). Table 1 further shows that the dates of birth in these samples were distributed unequally across the interval between 1400 and 1915, with progressively more composers and creative writers having been born in later centuries. Both the composer sample and the creative writer sample consisted of a mix of nationalities, though, unsurprisingly in light of the nature of our source, the majority of composers and creative writers were British.

**Table 1:** Characteristics of composers and creative writers in the study

	Composers	Creative writers
**Subjects (n)**	1110	1182
*Women (n (%))*	13 (1.2%)	182 (15.4%)
**Birth year distribution (n (%))**		
*1400 – 1499*	57 (5.1%)	3 (0.3%)
*1500 – 1599*	203 (18.3%)	52 (4.4%)
*1600 – 1699*	193 (17.4%)	114 (9.6%)
*1700 – 1799*	226 (20.4%)	238 (20.1%)
*1800 – 1915*	431 (38.8%)	775 (65.6%)
**Age at death in years (mean (sd))**	67.4 (13.1)	69.4 (13.9)
**Age at first work in years (mean (sd))**	23.3 (5.4)	28.8 (5.8)
**Nationality (n (%))**		
*Austria*	51 (4.6%)	4 (0.3%)
*Britain*	237 (21.4%)	719 (60.8%)
*France*	158 (14.2%)	82 (6.9%)
*Germany*	184 (16.6%)	31 (2.6%)
*Ireland*	-	66 (5.6%)
*Italy*	208 (18.7%)	29 (2.5%)
*Russia*	39 (3.5%)	32 (2.7%)
*Scotland*	1 (0.1%)	54 (4.6%)
*USA*	62 (5.6%)	97 (8.2%)
*Other countries*	170 (15.3%)	68 (5.8%)

**Figure 1:**
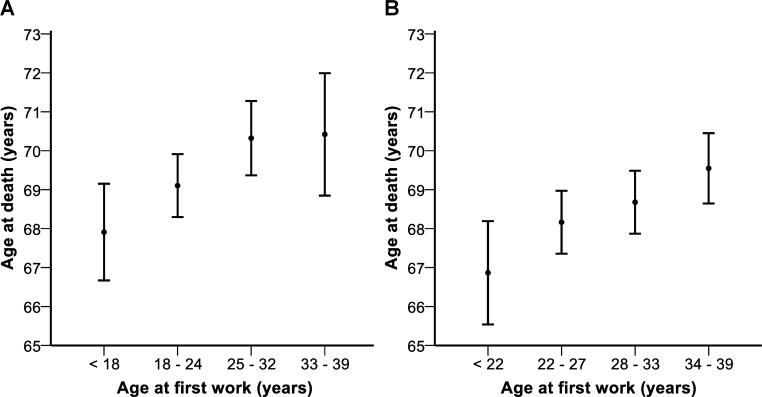
Age at first work and age at death for composers (A) and creative writers (B) Age at death per interval of age at first work was estimated as the marginal mean from a multilevel regression model that adjusted for sex, date of birth and nationality. Error bars denote 1 standard error.

Figure 1 shows age at death for various intervals of age at first work in both composers and creative writers. In both the composer sample and the creative writer sample, individuals with a later age at first work were found to have a higher average age at death. As shown in the figure, age at death increases from the earliest to the latest interval of age at first work with about 2.5 years in the composer sample and 2.7 years in the writer sample. The estimates shown in the figure were derived from a multilevel model that included the fixed variables age at first work, year of birth as an exponential term, and sex, as well as a random intercept for nationality. This model was parsimonious and fit the data well. Fitting the model with age at first work as a continuous variable instead of a variable grouped by intervals revealed a positive linear trend for age at death in both the composer sample (age at death 0.16 years higher for each later year of age at first work, *p* = 0.02) as well as the creative writer sample (age at death 0.18 years higher for each later year of age at first work, *p* < 0.01).

## DISCUSSION

In our study composers and creative writers with a later age at first work were shown to have a longer lifespan, when adjusting for sex, date of birth and nationality. To our knowledge, we are the first to demonstrate an association between the age at onset of intellectual maturity and age at death in humans.

### Strengths and limitations of the study

We believe one of the study's strengths is the demonstration of similar relationships between age at first work and age at death in two distinct populations of creative people. This reinforces the notion that the pattern has a biological underpinning and is not simply a peculiarity of either composers or creative writers. Even within the population of creative writers the same association was found in the more numerous subclasses (i.e. novelists, poets), although this association did not reach significance in these smaller subgroups.

Another of the study's strengths is the long historical time period, ranging from 1400 to 1915, during which the composers and creative writers included in our study were born. This is because patterns that are stable across historical time and varying cultural circumstances are more likely to have a biological underpinning and music and literature are ultimately products of an interplay between biology and culture. However, if left unaccounted for, historical trends present in both career course and mortality may confound associations between age at first work and age at death and invalidate the inference that any observed associations are biological in nature. Though not much is known about changes in the general course of musical or writing careers between 1400 and 1915, the changes in life expectancy in that time period have been relatively well-documented and led us to control for date of birth in our model. The result, we believe, is more robust evidence of an association between age at first work and age at death.

When interpreting our findings, it should be taken into consideration that our study samples do not represent randomly selected samples of composers and creative writers, but rather selections of creative historical individuals that the editors of the Oxford Companion series have deemed noteworthy. Selection may have also been at play at the level of the type or genre of the creative output. For instance, what constitutes music in the minds of the editors of the Companion to Music seems to be limited to classical music, as evidenced by the omission of performers and songwriters from more modern musical genres (e.g. jazz or pop music). The selection of classical composers by the editors of the Companion to Music and our exclusion of those born after 1915 might have given rise to a somewhat conservative picture of the lifestyles of those involved in music: contemporary musicians from the genres pop, rock, hip-hop and R&B have been shown to have a markedly higher mortality throughout their 20′s and 30′s than the general public [14, 15]. Although we do not have reasons to believe *a priori* that selection would distort our view of the association of interest, it warrants caution as we attempt to generalize our observations.

Another point worthy of consideration is that it is unclear whether we are justified in interpreting age at first work as a proxy for the onset of a general intellectual maturity. Though intellectual works must possess some level of quality to be appreciated by the public and by critics, both contemporary and future, it is not immediately clear whether the intellectual abilities involved in composing and writing have assumed an evolutionarily relevant level when the first work is made public. Neither is it clear how the timing of crossing this intellectual quality threshold maps onto the lifespan courses of fluid and crystallized intelligence – whether, for instance, it tracks the peak in one of the known dimensions of these two classes of intelligence [9, 12, 13].

If cultural factors explain the association reported here, the inference that the observed relationship is a biological one might be invalid. Therefore, it is worth considering whether such factors might exist. In the present study, socio-economic status, it stands to reason, might act as a classic confounder: composers and creative writers from richer families might have had more opportunities to work on creative enterprises early in life and would also be expected to live longer. Such associations, however, act to produce an inverse relationship between age at first work and age at death, and, if they are in effect in our study, will thus have dampened the magnitude of the association, apparently without being strong enough to abolish it altogether.

### Interpretation in view of life history theory

It would seem intuitive to define intellectual development as that part of the lifespan in which ability increases and intellectual maturity as that part in which ability plateaus or decreases. In such a model, an inflection point or peak in ability would mark the onset of intellectual maturity. It is unlikely, however, that the multidimensional abilities of composing and writing reach their peaks when a composer's or writer's work is made public for the first time. Rather, we regard maturity as a state that is attained when a certain relevant threshold is crossed, regardless of whether ability increases after that point. This not only conforms best to the usage of the term ‘maturity’ in common parlance (cf. 18 as the age at which persons are considered ‘mature enough’ to vote), but is also a sensible concept in an evolutionary context. Thus, although it is not clear whether becoming mature enough to see one's first creative outing made public corresponds to an evolutionarily relevant kind of maturity, we believe the general concept of intellectual maturity is valid in the context of life history theory.

Taking this view of maturity, the association reported here bears similarities to patterns in other organ systems reported elsewhere. As a hallmark of the onset of reproductive maturity, menarche at a later age has been shown to be correlated with higher longevity [4–6]. Similarly, a later age at physical peak has been shown to be associated with a later age at death [8]. Taken together, these lines of evidence of a common pattern in three distinct human organ systems (i.e. reproductive, locomotor and central nervous system) are suggestive of a broad underlying mechanism coupling age at onset of maturity and age at death, by linking rate of development to maintenance and repair.

The nature of this mechanism, however, remains elusive. From a life history perspective, it may be either a genetic mechanism or plasticity that couples rate of development and maintenance and repair. In the former scenario, genes that alter the rate of development such that maturity is reached earlier, would have as a concomitant effect the downregulation of maintenance and repair, leading to a shorter lifespan. Such mechanisms can serve to explain associations between development and longevity both within and between species. Classic r/K selection theory, which describes animals’ life histories in terms of the degree to which they are *r*-selected (many offspring, short lifespan) or *K*-selected (few offspring, long lifespan), serves as an inter-species example of concomitant variation of life history characteristics that is explained by genetic make-up [3, 16]. If it is plasticity that couples rate of development and maintenance and repair, it may be through adjustment of both the developmental program and maintenance and repair based on early-life environmental cues, so as to fit projected circumstances. In the specific case of intellectual development, plasticity as a coupling mechanism is implausible, since affluent early-life conditions that induce a faster rate of development would likely also benefit longevity, producing an association that runs counter to the one we observed.

### Generalizability to other intellectual behaviors

On the topic of generalizability, it is worth considering that composers and creative writers are at the extremes of spectrums of musical ability, language ability, and creativity, which some might consider specialist abilities. Consequently, composers and creative writers might seem to constitute groups whose defining intellectual products poorly represent general intellectual ability. However, rather than being unidimensional abilities, successful composition and successful writing likely require mastery of a whole suite of different intellectual abilities. Whether our results are generalizable to other complex, evolutionarily relevant intellectual behaviors thus depends on the generality of the abilities required for composing and writing and on the degree to which they are correlated with unexamined abilities. It is nearly self-evident that the prerequisite abilities for composition and writing should consist of abilities that are general to most intellectual behaviors (e.g. memory) as well as others rather specific to composing and writing (e.g. auditory pattern recognition in the case of composing and language abilities in the case of writing). Where composing is concerned, musical ability has been shown to correlate with general intelligence as well as more specific abilities, such as visuospatial abilities [17, 18]. Additionally, levels of many other distinct intellectual abilities have been shown to be positively correlated, which has provided the basis for the construct of general intelligence in the first place [19]. From this perspective, composing and writing seem to chiefly represent either specialized applications or strong correlates of rather general intellectual abilities that are likely to be present in a host of other evolutionarily relevant intellectual behaviors.

## CONCLUSION

The results of this study support the notion that the onset of intellectual maturity is associated with longevity, as has been previously demonstrated for the onset of reproductive and physical maturity. Given the prominence of intelligence in humans’ evolutionary history, further elucidating its relation to other life history characteristics would greatly advance our understanding of the evolution of our species.

## MATERIALS AND METHODS

This study was performed using historical biographical data on musical composers and creative writers.

### Data source

The data used for the present study were courteously provided by William Bains. A more detailed description of the dataset and its compilation can be found in his initial publication [20]. In short, the dataset provided to us had been compiled by manual extraction of biographical information from The Oxford Companion to Music [21], which contains historical information on musical composers and musicians, and The Oxford Companion to English Literature [22], which contains historical information on creative writers and others involved in publishing. The information gathered had been checked against online databases and inconsistencies had been resolved by cross-referencing Baker's Biographical Dictionary of Musicians [23] or The Wiley-Blackwell Encyclopedia of Literature [24], where they concerned composers or creative writers respectively. For each composer and each writer included in the analysis the following characteristics were known: name, sex, date of birth, date of death, date of first work and nationality.

### Criteria for in- or exclusion

Both datasets contained individuals that we excluded from our analyses: of the individuals involved in music we excluded those that had solely performed music composed by others; of the individuals involved in literature we excluded those that had not created original work, such as translators and publicists. Those who had indeed written original work, viz. creative writers, had been classified into several non-mutually exclusive subclasses: novelists, poets, playwrights/dramatists, humorists/satirists, non-fiction writers, screen writers/radio writers, scholars/philosophers, journalists or diarists/memoirists.

Additionally, we excluded from our analyses all individuals whose first work had come out after the age of forty or who had died before the age of forty. As a result, the intervals in which either event took place did not overlap in our final study populations. Such measures were necessary to avoid a spurious positive correlation between age at first work and age at death, which would arise from the fact that, in order to have even been included in our dataset as a composer or a writer, individuals who completed their first work at a late age, would necessarily have died even later, while individuals who died early, would have to have completed their first work at an earlier age.

Finally, the dates of birth of the composers and creative writers included in our datasets ranged from the year 840 AD to the year 1971 AD. However, more recent birth cohorts have not yet experienced complete mortality and thus add to analyses of lifespan only individuals who have died at an uncharacteristically young age. Therefore, in order to avoid spurious associations between date of birth, age at death and age at first work, composers and creative writers born after 1915 were excluded. Furthermore, composers and creative writers born before the year 1400 were also excluded, for reasons of a paucity of records on individuals born before this date and a lower reliability of estimates of date of birth and date of death that lie before this date.

### Statistical analysis

The association between age at first work and age at death was modelled using multilevel regression, with nationality as a higher-level grouping variable, adjusting for sex and year of birth. Two separate models were fit: one with age at first work as a variable grouped by intervals and one with age at first work as a continuous variable, so as to test for a linear trend. The intervals, by which values of age at first work were grouped, were constructed by dividing the observed range of values for age at first work into four parts of similar width for composers and creative writers separately. All statistical analyses were performed using SPSS Statistics 24.0 (IBM Corp., Armonk, NY).

## References

[1] Roff DA (1992). The evolution of life histories: theory and analysis.

[2] Rollo CD (2002). Growth negatively impacts the life span of mammals. Evol Dev.

[3] Kaplan H, Hill K, Lancaster J, Hurtado AM (2000). A theory of human life history evolution: diet, intelligence, and longevity. Evol Anthropol.

[4] Jacobsen BK, Heuch I, Kvåle G (2007). Association of low age at menarche with increased all-cause mortality: a 37-year follow-up of 61,319 Norwegian women. Am J Epidemiol.

[5] Jacobsen BK, Oda K, Knutsen SF, Fraser GE (2009). Age at menarche, total mortality and mortality from ischaemic heart disease and stroke: the Adventist Health Study, 1976-88. Int J Epidemiol.

[6] Lakshman R, Forouhi NG, Sharp SJ, Luben R, Bingham SA, Khaw KT, Wareham NJ, Ong KK (2009). Early age at menarche associated with cardiovascular disease and mortality. J Clin Endocrinol Metab.

[7] Westendorp RG, Kirkwood TB (1998). Human longevity at the cost of reproductive success. Nature.

[8] Van de Vijver P, Van Bodegom D, Westendorp RG (2016). Early and extraordinary peaks come with a longevity cost. Aging (Albany NY).

[9] Germine LT, Duchaine B, Nakayama K (2011). Where cognitive development and aging meet: face learning ability peaks after age 30. Cognition.

[10] Cattell RB, Mifflin Houghton (1971). Abilities: Their structure, growth, and action.

[11] Baltes PB (1987). Theoretical propositions of life-span developmental psychology: on the dynamics between growth and decline. Dev Psychol.

[12] Craik FI, Bialystok E (2006). Cognition through the lifespan: mechanisms of change. Trends Cogn Sci.

[13] Hartshorne JK, Germine LT (2015). When does cognitive functioning peak? The asynchronous rise and fall of different cognitive abilities across the life span. Psychol Sci.

[14] Bellis MA, Hennell T, Lushey C, Hughes K, Tocque K, Ashton JR (2007). Elvis to Eminem: quantifying the price of fame through early mortality of European and North American rock and pop stars. J Epidemiol Community Health.

[15] Wolkewitz M, Allignol A, Graves N, Barnett AG (2011). Is 27 really a dangerous age for famous musicians? Retrospective cohort study. BMJ.

[16] Pianka ER (1970). On r-and K-selection. Am Nat.

[17] Lynn R, Gault A (1986). The relation of musical ability to general intelligence and the major primaries. Res Educ.

[18] Hassler M, Birbaumer N, Feil A (1985). Musical talent and visual-spatial abilities: A longitudinal study. Psychol Music.

[19] Humphreys LG (1979). The construct of general intelligence. Intelligence.

[20] Palacios T, Solari C, Bains W (2015). Prosper and Live Long: Productive Life Span Tracks Increasing Overall Life Span Over Historical Time among Privileged Worker Groups. Rejuvenation Res.

[21] Latham A (2002). The Oxford companion to music.

[22] Drabble M (2006). The Oxford companion to English literature.

[23] Slonimsky N, Kuhn LD, Centennial (2001). Baker's biographical dictionary of musicians.

[24] Day G, Lynch J, Felluga DF, Hughes W, Punter D, Smith A, Sullivan GA, Stewart A, Burwick F, Moore Goslee N, Long Hoeveler D, Shaffer BW (2013). Wiley-Blackwell Encyclopedia of Literature.

